# Pharmacotherapy of eating disorders during transition from adolescence to adulthood

**DOI:** 10.1192/j.eurpsy.2026.10388

**Published:** 2026-07-31

**Authors:** H. Papežova

**Affiliations:** Psychiatric Department, First Medical School and University Hospital, ED Unit, Charles University Prague, Czech Republic

## Abstract

**Background:**

The transition from adolescence to adulthood represents a critical developmental period associated with increased vulnerability to the persistence or worsening of eating disorders (EDs). Pharmacotherapy, though not first-line for most EDs, plays a complementary role in addressing core symptoms, psychiatric comorbidities, and medical complications.

**Objectives:**

To summarize current evidence on the efficacy, safety, and clinical considerations of pharmacological treatments for anorexia nervosa (AN), bulimia nervosa (BN), and binge‑eating disorder (BED) during the transition age (approximately 16–25 years).

**Methods:**

A narrative synthesis of clinical trials, recent systematic reviews and guideline recommendations with established or emerging evidence..

**Results:**

Evidence for pharmacotherapy in transitional-age patients remains limited. For AN, no medication has robust efficacy in weight restoration; however, atypical antipsychotics—particularly olanzapine—may support weight gain and reduce cognitive rigidity, though metabolic risks require close monitoring. In BN, selective serotonin reuptake inhibitors (SSRIs), especially fluoxetine at higher doses reduce binge–purge frequency and mood symptoms. BED pharmacotherapy is better supported: lisdexamfetamine demonstrates significant reductions in binge frequency, while topiramate reduce binge episodes and weight but with cognitive side‑effect risks. Comorbid anxiety, depression, and obsessive–compulsive symptoms often guide adjunctive medication. Developmental considerations are essential in treatment.

**Conclusions:**

Pharmacotherapy can play a valuable adjunctive role in treating ED during the transition from adolescence to adulthood. Evidence remains insufficient for AN. Careful monitoring for metabolic, cardiovascular, and neuropsychiatric adverse effects is crucial. Integrated, developmentally informed models of care. Supporting continuity between child/adolescent and adult services are needed to optimize medication. Further controlled trials are required.

**Literature:**

HIMMERICH, H. et al.:The WFSBP Task Force on Eating Disorders. WFSBP guidelines update 2023 on the pharmacological treatment of eating disorders. The World Journal of Biological Psychiatry. 2023. DOI: 10.1080/15622975.2023.2179663.

Supported by Ministry of Health, CR NU23-04-00381, Cooperatio Program (Neuroscience and Health Sciences).

**Disclosure of Interest:**

None Declared

